# Editorial: Skin: benefits of natural products in topical applications

**DOI:** 10.3389/fphar.2023.1305705

**Published:** 2023-11-10

**Authors:** Diana Simona Antal, Ștefana Avram, Michael Heinrich

**Affiliations:** ^1^ Department of Pharmaceutical Botany, Faculty of Pharmacy, Victor Babes University of Medicine and Pharmacy Timisoara, Timisoara, Romania; ^2^ Department of Pharmacognosy, Faculty of Pharmacy, Victor Babes University of Medicine and Pharmacy Timisoara, Timisoara, Romania; ^3^ UCL School of Pharmacy, University College London, London, United Kingdom; ^4^ Chinese Medicine Research Center, Department of Pharmaceutical Sciences and Chinese Medicine Resources, College of Chinese Medicine, China Medical University, Taichung, Taiwan

**Keywords:** natural products, topical application, atopic dermatitis, wound healing, skin photoaging

The skin is the largest human organ, acting as an essential barrier towards the external environment. It provides protection against various traumatic events, and serves moreover as temperature and fluid controller (Shedoeva et al., 2019). The extended exposure of the skin contributes to its vulnerability, thus leading to various dermatologic conditions, highly impacting the welfare of humans, causing important burden on the healthcare systems (Ajjoun et al., 2022).

The management of skin disorders is inspired by traditional practices using medicinal plants and metabolites (natural products) derived from them as therapeutic approaches from ancient times to the present day. Plant extracts are renowned for their pleiotropic effects. Applied to the skin, they can provide versatile pharmacological tools that restore the cell-to-cell signaling in diseases like a Research Topic dermatitis and psoriasis, influence mechanisms involved in skin cancer, exert anti-inflammatory, antioxidant, antimicrobial, wound-healing and anti-aging effects (Mohd Zaid et al., 2022).

Both isolated metabolites, and plant extracts are important in treating skin diseases. Recent advances in natural products research added valuable mechanistic insights for traditional medicines. Moreover, on a global scale, natural products represent a valuable alternative, owing to their accessibility, safety and low cost (Malik et al., 2019). The development of current topical preparations focuses on the characterization of natural compounds composition, the combination with modern drugs, as well as innovative delivery systems.

This Research Topic resulted in six articles bringing together experimental and review papers that explore the benefits of topical application of natural extracts on various skin conditions. The major themes within this topic included restoration of atopic dermatitis symptoms, management of wound healing process and tackling skin aging.

Wing Sze Lai et al. focused on a traditional popular Asian food supplement, Edible bird’s nest (EBN), a popular delicacy representing a salivary secretion from several *Aerodramus* spp. swiftlets, mainly *Aerodramus fuciphagus*. The study analyzed the potential anti-inflammatory activity of EBN extracts with specific benefits in atopic dermatitis. The enzymatic digest of EBN showed good anti-inflammatory responses both *in vitro* and *in vivo* assessments. The authors indicate the possible EBN -mediated anti-inflammatory response: inducing expression of filaggrin and filaggrin-2, reduction of reactive oxygen species (ROS), NF-κB signaling and phosphorylation of p38, MAPK and JNK. Moreover, the enzymatic digest of EBN impacted on skin thickness, the severity level of damage and scratching behavior, *in vivo*. Thus, the study underlines the multiple advantages of EBN-derived products in the management of atopic dermatitis, such as bioavailability, costs and low toxicity.

Fan Y et al.researched the protective effects of extracts from *Acer truncatum* Bunge leaves (ATLE) on a dermatitis model using SLS-induced HaCaT cells. The extract induced increased viability and survival, reducing apoptosis in SLS-stimulated HaCaT cells, while downregulating the expression of PGE2 and IL-6 proinflammatory factors, hence protecting the cells from SLS-caused damage. Four flavonoids with proven anti-inflammatory effects were isolated and identified from *A. truncatum* leaves, kaempferol-3,7-di-O-α-L-rhamnoside, being firstly reported in this species.

Bhat P et al. investigated a topical ointment containing phenol enriched fraction (PEF) of *Caesalpinia mimosoides* Lam. for potential use in wound therapy. The plant is an important remedy used in the treatment of skin ailments by traditional practitioners in India. Potent wound healing activity of PEF was reported, with high concentrations of gallic acid and ethyl gallate. The *in vitro* biological evaluation of the PEF showed an antioxidant capacity similar to ascorbic acid, an anti-inflammatory effect comparable to diclofenac sodium, and a broad-spectrum antimicrobial, while effectively stimulating the skin cell migration rates. A significantly shorter wound closure was noticed in 5%, better than that of standard Povidone-Iodine was evidenced, *in vivo*. In this study, the ointment containing PEF of *C. mimosoides* was shown to accelerate the wound healing process and potentially shortening the healing time.

Another work from this Research Topic addressed the management of skin wound healing. Paul-Traversaz M et al. reviewed the existing data regarding the active ingredients of three important herbal ointments for skin wound healing in Kampo traditional medicine. As a first attempt in the scientific literature, the authors focused on the chemical composition and mechanisms of action of Japanese Kampo ointments, namely, *Shiunkō*, *Chuōkō*, and *Shinsen taitsukō*. The bibliographic work highlighted the difficulties considering the chemical and biological analysis of lipophilic extracts, and revealed valuable data regarding lipophilic secondary metabolites from species contained in the Kampo ointments such as curcumin, shikonin, imperatonin, and byakangelicin that could be involved in the management of wound healing process, exerting anti-inflammatory, antiseptic, antioxidant and protective effects.

Ma L. et al. focused on the protective effect of Puerariae Lobatae Radix (*Pueraria montana* var. *lobata* (Willd.) Maesen & S.M.Almeida ex Sanjappa & Predeep, PLR) topical application against UVB-induced skin aging on mice. The authors investigated the mechanistic insights involved, proving that PLR reduced skin wrinkles, epidermal thickness, and malondialdehyde content as well as increased levels of hydroxyproline and superoxide dismutase, decreasing *Mmp-1*, *p21* and *p53* levels in the skin. Also, PLR upregulated skin expression of *BMAL1*, an aging-inhibiting factor, by promoting *Nrf2* and antioxidant enzymes. Additionally, PLR acted as an antagonist of *REV-ERBα*, which protected mice against UVB-induced skin aging, indicative for the control of photoaging.

Another study explored the potential effects against skin aging. Gu M J et al. aimed to investigate the mechanism of *Schizonepeta tenuifolia* Briq. (ST) extract involved in the prevention of skin photoaging in UVB-irradiated HR-1 mice. The expression of matrix metalloproteinases was reduced, while tissue inhibitor of metalloproteinase 1 expression was upregulated. ST application also improved skin dehydration, decreasing levels of hyaluronidase-1 and -2 and increasing the expression of hyaluronan synthases and hyaluronic acid levels. UVB-induced skin damage was considerably attenuated by regulating the expression of MAPKs, accumulation of AGE and expression of the receptor for AGE, hence, endorsing ST as a potential candidate for anti-photoaging management.

This Research Topic includes a collection of articles on potential benefits of topical application of natural products candidates in atopic dermatitis, wound healing and skin photoaging, contributing with valuable data and mechanistic insights to a better therapeutic management of these skin conditions. It highlights achievements in the field, but also points to the great need for more systematically exploring the potential of medicinal plants in the treatment of inflammatory and infectious skin conditions. There is a great unmet need and the potential deserves a wider and systematic scientific attention.
